# Real world outcomes of the new Tecnis Eyhance IOL

**DOI:** 10.1177/11206721221146675

**Published:** 2022-12-25

**Authors:** Edward Gigon, Walid Bouthour, Georgios D Panos, Bojan Pajic, Horace Massa

**Affiliations:** 1 Department of Clinical Neurosciences, Division of Ophthalmology, Geneva University Hospitals, Geneva, Switzerland; 2 Department of Ophthalmology, Queen‘s Medical Centre, 9820 Nottingham University Hospitals NHS Trust, Nottingham, UK; 3 Eye Clinic Orasis, Swiss Eye Research Foundation, Reinach AG, Switzerland; 4 Faculty of Medicine, University of Geneva, Geneva, Switzerland; 5 Faculty of Sciences, Department of Physics, University of Novi Sad, Novi Sad, Serbia; 6 Faculty of Medicine of the Military Medical Academy, University of Defense, Belgrade, Serbia

**Keywords:** IOLs, clinical tests, IOL calculation for primary / secondary IOL, phacoemulsification, biometry / axial length

## Abstract

**Purpose:**

To compare the performance of Tecnis Eyhance ICB00 with Tecnis PCB00 IOL for far, intermediate, and near vision, in patients after bilateral cataract surgery.

**Settings:**

This study was done at Geneva University Hospitals.

**Design:**

This is a retrospective study of 224 eyes that underwent cataract between May 2019 and June 2020.

**Methods:**

Visual acuity was assessed from month 1 to 12 after surgery for distance, intermediate and near visual acuity, by the same optometrist, which was blind regarding the type of IOL. The patients answered to a quality of life questionnaire. Patients were excluded for: monocular surgery, macular disease, other IOL type, or inability to reach 20/20 visual acuity in both eyes without correction.

**Results:**

One hundred and fifty-two eyes were excluded. Three groups were then analyzed: PCB00 group (38 eyes), ICB00 group (22 eyes), and mismatch group (12 eyes). Monocular visual acuities (CIVA, UNVA and CNVA, in logMAR) were higher in the ICB00 group than the PCB00 group (respectively 0.3 vs 0.4, p = 0.0033; 0.3 vs 0.4, p = 0.0408; 0.3 vs 0.4, p = 0.0039). Binocular visual acuities, CIVA and CNVA were higher in the ICB00 group than the PCB00 group (0.2 vs 0.4, p = 0.0061; 0.15 vs 0.3, p = 0.018). This mirrored the findings of the quality of life questionnaire. There was no significant difference between PCB00 and mismatch groups.

**Conclusions:**

the Tecnis Eyhance was more effective for intermediate and near vision. The central defocus of the lens might help patients achieve spectacle independence and better quality of life.

## Introduction

Cataract surgery is one of the most common surgical procedures worldwide.^1^ Despite continuous research and efforts to achieve perfect refractive results, patients may still experience a disappointing outcome.^2^ This is mainly due to an unexpected refractive surprise, and sometimes due to the patients’ expectations as well.^3^ With increasing post-operative patients’ requirements, another cause of post-operative dissatisfaction is the loss of intermediate vision. Patients over 60 years usually get used to presbyopia, but intermediate vision is somewhat preserved. Switching from a spherical intra ocular lens (IOL) to an aspherical one may help improve contrast sensitivity in mesopic or photopic conditions.^4^ Aspherical IOLs also increase the best corrected visual acuity and have better visual quality regardless of distance.^3^

Alternative options like the Extended Depth of Focus (EDOF), or multifocal IOLs, were designed to restore intermediate and near vision, but are not suitable for all patients. Halos and glares are common complications after trifocal IOLs implantation and patients need usually a few months to get used to multifocal IOLs.^5^ Unfortunately, poor neuroadaptation sometimes occurs and some of these patients need an IOL exchange, which is a challenging surgery.^2^ Careful patient selection is therefore crucial. Another issue is that these IOLs are also relatively expensive, which limits their widespread use.^6^

As smartphones, tablets and computers are becoming extremely common in everyday life; intermediate vision has become even more important than distance vision in daily activities of patients.^7^ For the above reasons, the need arose to switch from bifocal to trifocal IOLs, and since not all patients were eligible for such IOLs, another IOL, the Tecnis Eyhance IOL, was brought to the market (Johnson&Johnson Vision, Santa Ana, CA USA). The Tecnis Eyhance is a monofocal IOL with a little increased depth of focus due to a modified anterior aspheric surface, which, in theory, leads to a progressive defocus up to 1 dioptre (D).^8^

In this study, we aim to assess near and intermediate vision and compare the Tecnis Eyhance ICB00 IOL with the monofocal Tecnis PCB00 IOL (Johnson&Johnson Vision, Santa Ana, CA USA) in patients that remained spectacle-free for distance vision after cataract surgery.

## Methods

This is a retrospective study of 224 eyes that underwent cataract surgery at Geneva University Hospitals. All cases were operated by the same surgeon (H.M.) between May 2019 and June 2020.

Surgery was performed using topical or general anesthesia with the main incision in the axis of the steepest corneal curvature and one 20G paracentesis. Capsulorhexis was generally small, about 4.5 mm and the lens delivery was done using mostly the divide-and-conquer technique with the Centurion phaco machine (Alcon Laboratories, Switzerland). Injection of the IOL was done following the instruction manuals without penetrating into the anterior chamber. At the end of the surgery, incisions were hydrated and 0.1 mL of cefuroxime was injected into the anterior chamber. Post-operative regimen was the same for all patients, a topical combination of moxifloxacine during the first week and an association of tobramycine and dexamethasone for 3 weeks followed by bromfenac for 2 weeks.

Only patients with bilateral surgery with one of the following implant type were included: Tecnis PCB00 IOL or Tecnis Eyhance ICB00 IOL. No particular condition was associated with the choice of the IOL type, except patients with obvious macular disease where the ICB00 IOL was not considered, or patient with an important astigmatism (>2 D). IOL power was determined based on the results of IOL Master 700 biometry device (Carl Zeiss Meditec, Jena, Germany) using the SRK/T formula so as to obtain a post-operative refraction as close as possible to emmetropia.

Patients were excluded from analysis if distance Snellen visual acuity could not reach at least partially 10/10 Snellen on both eyes for distance vision at 5 metres or if surgery was performed on only one eye as we only included spectacle free patients. Patients with macular impairment were excluded.

At the end of the patient selection protocol, four different patient groups remained: the excluded patient group, the ICB00 IOL group with one ICB00 IOL in each eye, the PCB00 group with one PCB00 lens in each eye, and the mismatch group with PCB00 lens in one eye, and ICB00 lens in the other.

All patients signed an institutional consent form postoperatively in order to be included in the statistical analysis, as per the Declaration of Helsinki.

All post-operative visual acuity measurements were done by the same qualified optometrist, in the same room, with the same photopic conditions for distance, intermediate (65 cm) and near (35 cm) visual acuity. Visual acuity was assessed with the visutron 900 from Haag-Streit (Bern, Switzerland) using a polarized screen for distance and a reading table for near and intermediate visual acuity with the same additional reading light.

Visual acuity measurements were performed without correction and with distance correction. Refraction was assessed in monocular fashion and then binocular fashion. The optometrist was blind to patients’ IOL selection. Visual acuities were prospectively assessed on a study form. Postoperative visual acuity was assessed at least one month after surgery and up to one year. Visual acuity measurements were retrieved in Snellen and then converted to the logarithm of the Minimal Angle of Resolution (logMAR) for statistical analysis.

Population, eyes and post-operative characteristics were recorded based on the electronic medical record and data from the IOL master 700. The ophthalmologist also collected patients’ satisfaction using the PRISQ questionnaire (Patient-Reported spectacle Independence Questionnaire).^9^

### Statistical analysis

We used the Shapiro-Wilk test to assess data normality. Groups were compared using one-way ANOVA or Student t-test. Whenever distribution was non-Gaussian, we used non-parametric Kruskal-Wallis test or Mann-Whitney test for skewed data. We used *X*2 test for contingency tables. Significance threshold was 0.05. We used GraphPad Prism (v9.3.1) for statistics and figures.

## Results

**Figure 1.**
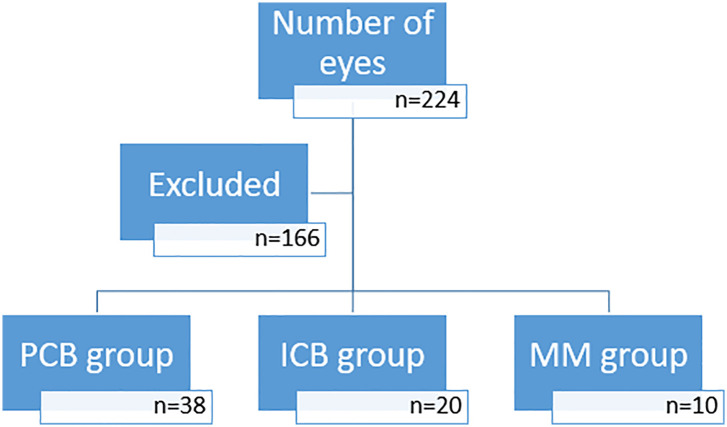
Chart view of the study.

We enrolled 224 cataract surgeries performed between May 2019 and June 2020 by the same surgeon (HM). One hundred and fifty-two eyes were excluded because of monocular surgery, significant macular disease, other type of IOL used, or could not reach at least a partial 1.0 Snellen visual acuity in both eyes without correction, or did not show up for intermediate and near visual acuity follow-up. Seventy-two eyes in 36 patients were subsequently included in this retrospective study, and fell into three groups: the PCB00 IOL group (38 eyes from 19 patients), the ICB00 IOL group (22 eyes from 11 patients), and the mismatch group (12 eyes from 6 patients) (Figure 1). These groups will henceforth be referred to as PCB00, ICB00 and MM group.

### Preoperative data

The population tended to be younger in the PCB00 group (median age 65 years) in comparison to the ICB00 group (median age 75.5 years) although the difference was not significant (Kruskal-Wallis one way, H(2) = 5.02, *p* = 0.081) (Table 1). Men were predominant in the PCB00 and MM groups while more women were in the ICB00 group, although the difference was not significant (Chi-square, *X*^2^ (2, N = 36) = 1.85, *p* = 0.396). (Table 1)

**Table 1.** Demographics and preoperative data values in the three groups.

	PCB	ICB	MM	Test result	*P value*
**Age (years, median)**	65	76	78.5	H(2) = 5.02	*0.081*
**Women (total)**	8 (19)	7 (11)	2 (6)	*X*^2^ (2, N = 36) = 1.85	*0.396*
**Axial length (mm, median)**	23.55	23.74	23.31	H(2) = 1.42	*0.491*
**Anterior chamber depth (median, mm)**	3.21	3.03	3.13	H(2) = 0.17	*0.917*
**Preoperative CDVA (median, logMAR)**	0.2	0.3	0.3	H(2) = 1.33	*0.514*
**Spherical equivalent (median, D)**	+ 0.63	0.00	−1.25	H(2) = 3.57	*0.168*
**Preoperative cylinder (median, D)**	−0.75	−0.50	−1.00	H(2) = 1.23	*0.540*
**Preoperative sphere (median, D)**	43.99	42.87	44.62	H(2) = 15.08	*0.001*
**Power IOL (median, D)**	22.00	22.50	21.50	H(2) = 0.32	*0.852*
**Target SRK/T (median)**	−0.44	−0.25	−0.30	H(2) = 13.89	*0.001*

D : dioptres; CDVA: corrected distance visual acuity; IOL : intraocular lens.

The median axial length was 23.55 mm, 23.74 mm and 23.31 mm in PCB00, ICB00 and MM groups respectively. There was no significant difference between groups (Kruskal-Wallis one way, H(2) = 1.42, *p* = 0.491). The median anterior chamber depth was 3.21 mm, 3.03 mm, and 3.13 mm respectively, and there was no significant between groups (Kruskal-Wallis one way, H(2) = 0.17, *p* = 0.917). (Table 1).

The median preoperative Corrected Distance Visual Acuity (CDVA) was 0.2, 0.3, and 0.3 LogMAR respectively, but there was no significant difference between groups (Kruskal-Wallis one way, H(2) = 1.33, *p* = 0.514) (Table 1).

Preoperative sphere values showed a tendency toward hyperopia in MM (median −1.25 dioptres) and PCB00 (median + 0.63 dioptres), and toward myopia in ICB00 (median 0.00 dioptre). Cylinder median values were −0.75, −0.50 and −1.00 dioptres in these respective groups. However, the sphere and cylinder values were not significantly different between groups (sphere: Kruskal-Wallis one way, H(2) = 3.57, *p* = 0.168; cylinder: Kruskal-Wallis one way, H(2) = 1.23, *p* = 0.540). (Table 1)

Corneal spherical equivalent was higher in MM group (median 44.62 dioptre) than in PCB00 (median 43.99 dioptre), and ICB00 (median 42.87 dioptre). The differences were significant (Kruskal-Wallis one way, H(2) = 15.08, *p* = 0.001) (Table 1).

IOL power was not significantly different between groups (median 22.00, 22.50, and 21.50 dioptres in respective groups; Kruskal-Wallis one way, H(2) = 0.32, *p* = 0.852) (Table 1). The target SRK/T was closer to emmetropia in ICB00 (median −0.25) while it tended to be more myopic in PCB00 (median −0.44) and in MM (median −0.30). These differences were significant (Kruskal-Wallis one way, H(2) = 13.89, *p* = 0.001). (Table 1).

### Postoperative data

#### Visual acuity data

**Figure 2.**
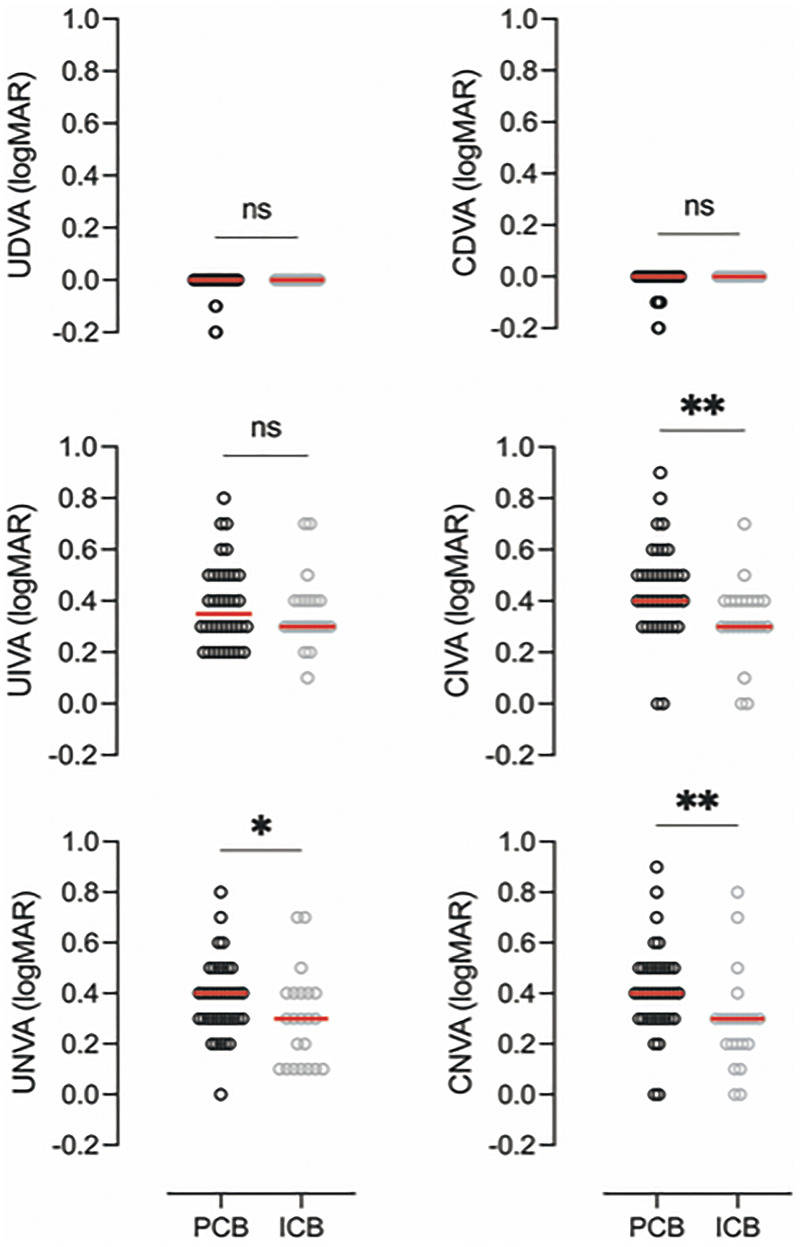
Monocular visual acuity repartition between the groups in LogMar scale. U/CDVA : un/corrected distance visual acuity, U/CIVA un/corrected intermediate visual acuity, U/CNVA un/corrected near visual acuity.

All patients had at least partial 0.00 LogMAR distance visual acuity in both eyes, thus our analysis is divided into two parts: with and without far correction (Figure 2).

The distribution of post-operative refraction in spherical equivalent (SE) was uniform in all groups, with a vast majority of patients falling between −0.50 and plano. (Supplementary file 1)

#### Monocular vision

**Figure 3.**
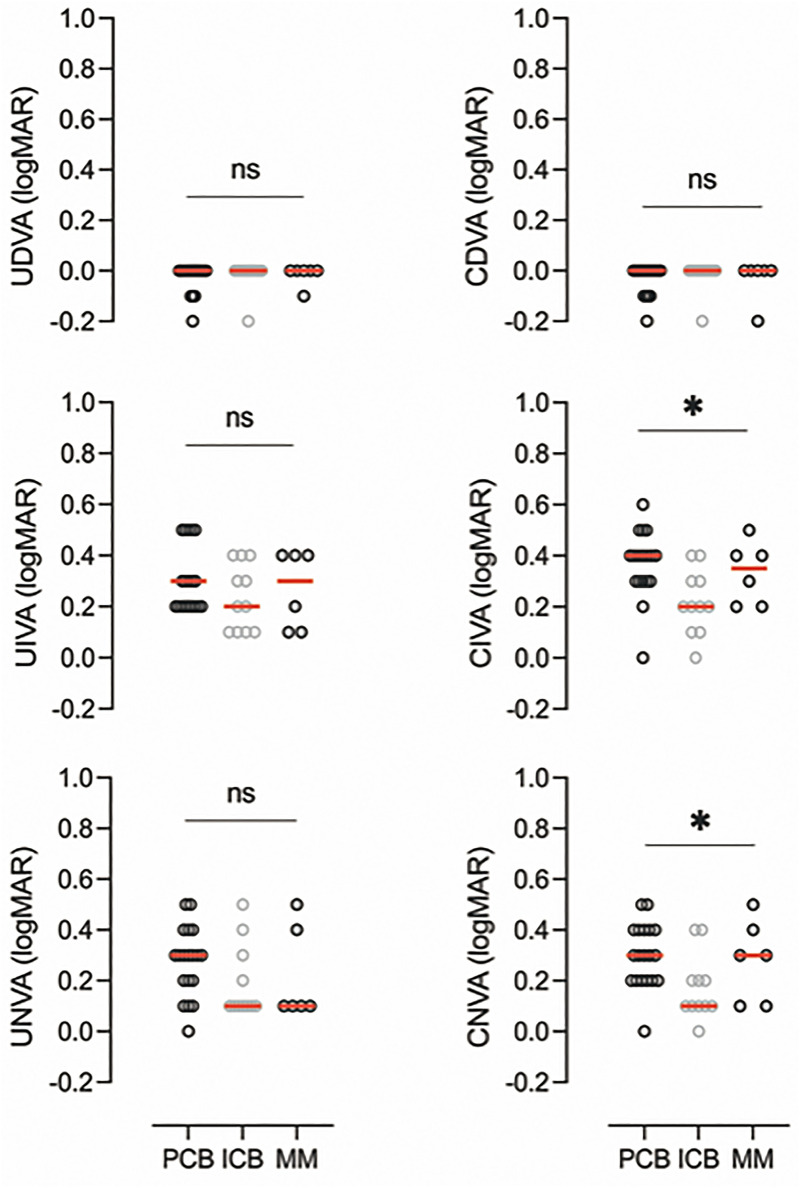
Binocular visual acuity repartition between the groups in LogMar scale. U/CDVA : un/corrected distance visual acuity, U/CIVA un/corrected intermediate visual acuity, U/CNVA un/corrected near visual acuity.

**Distance vision**. Visual acuity was at least 0.00 in LogMAR scale in PCB00 and ICB00 without correction. There was no significant difference between groups (Mann-Whitney U test, U = 374, *p* = 0.286). (Figure 3).

**Intermediate vision**. Visual acuities without correction were not significantly different in PCB00 (0.35 LogMAR) compared to the ICB00 (0.30 LogMAR) (Mann-Whitney U test, U = 385.5, *p* = 0.613). This difference became statistically significant with the use of an optic correction for distance vision, with a visual acuity of 0.4 LogMAR in PCB00 and 0.3 LogMAR in ICB00 (Mann-Whitney U test, U = 238, *p* = 0.004) (Figure 3).

**Near vision**. Visual acuity without distance correction was 0.4 LogMAR in PCB00, and 0.3 LogMAR in ICB00, and the difference was statistically significant (Mann-Whitney U test, U = 288, *p* = 0.041). These differences remained statistically significant with distance correction (Mann-Whitney U test, U = 216.5, *p* = 0.001) (Figure 3).

#### Binocular vision

**Figure 4.**
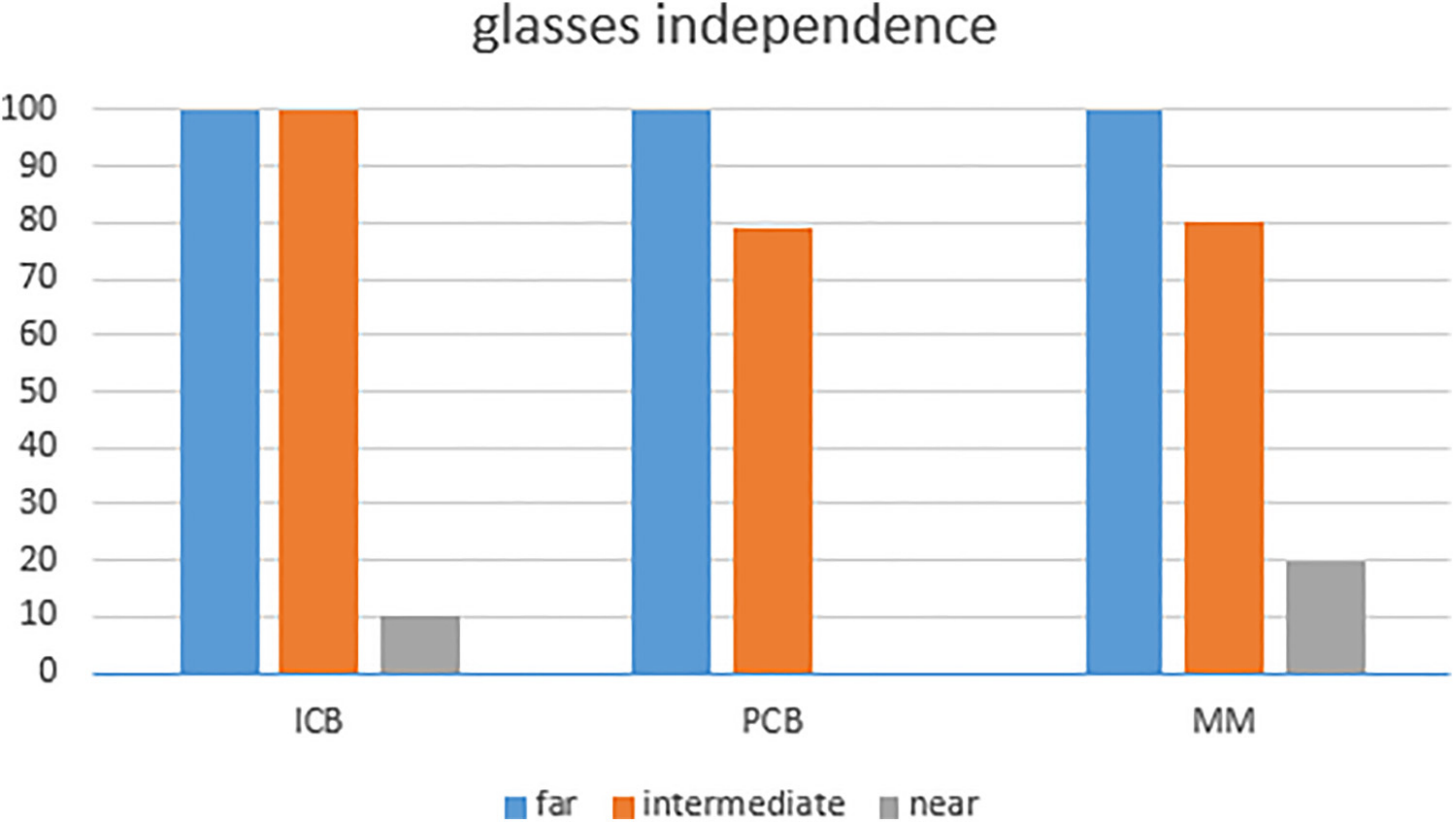
Glasses independence reported in the PRISQ.

After comparing corrected and uncorrected distance, intermediate, and near visual acuities in all groups, the only significant differences we found in binocular vision were in corrected intermediate visual acuity (CIVA) (medians 0.4 LogMAR in PCB00, 0.2 LogMAR in ICB00, and 0.35 LogMAR in MM; Kruskal-Wallis one way, H(2) = 8.64, *p* = 0.013*), and corrected near visual acuity (CNVA) (medians 0.3 LogMAR in PCB00, 0.1 LogMAR in ICB00, and 0.3 LogMAR in MM; Kruskal-Wallis one way, H(2) = 6.32, *p* = 0.043*).(Figure 4)

### Quality of life assessed with the PRISQ test

Results are reported in table 2.

**Table 2.** The PRISQ questionnaire results (patient-reported spectacle independence questionnaire).

ICB00 group (n = 11)		Cat 1	Cat 2	Cat 3	Cat 4	Cat 5
Did you need glasses for :	D	_	11 (100%)			
	I	_	11 (100%)			
	N	10 (90%)	1 (10%)			
How often did you wear glasses or contacts for:	D	_	_	_	1 (10%)	10 (90%)
	I	_	_	_	2 (20%)	9 (80%)
	N	4 (35%)	4 (35%)	2 (20%)	_	1 (10%)
Were you able to function comfortably without glasses or contacts for:	D	11 (100%)	_	_	_	_
	I	9 (80%)	2 (20%)	_	_	_
	N	_	2 (20%)	6 (55%)	_	3 (30%)

**Table:** 

PCB00 group (n = 19)		Cat 1	Cat 2	Cat 3	Cat 4	Cat 5
Did you need glasses for :	D	_	19 (100%)			
	I	4 (20%)	15 (80%)			
	N	19 (100%)	_			
How often did you wear glasses or contacts for:	D	_	_	_	_	19 (100%)
	I	1 (5%)	1 (5%)	2 (10%)	1 (5%)	14 (75%)
	N	8 (45%)	5 (25%)	5 (25%)	1 (5%)	_
Were you able to function comfortably without glasses or contacts for:	D	19 (100%)	_	_	_	_
	I	14 (75%)	3 (15%)	1 (5%)	1 (5%)	_
	N	_	3 (16%)	3 (16%)	10 (52%)	3 (16%)

**Table:** 

MM group (n = 6)		Cat 1	Cat 2	Cat 3	Cat 4	Cat 5
Did you need glasses for :	D	_	6 (100%)			
	I	1 (15%)	5 (85%)			
	N	5 (85%)	1 (15%)			
How often did you wear glasses or contacts for:	D	_	_	_	_	6 (100%)
	I	_	1 (15%)	_	1 (15%)	4 (70%)
	N	2(33%)	2 (33%)	2 (33%)	_	_
Were you able to function comfortably without glasses or contacts for:	D	6 (100%)	_	_	_	_
	I	4 (70%)	1 (15%)	1 (15%)	_	_
	N	_	1 (15%)	3 (50%)	1 (15%)	1 (15%)

D = distance; I = intermediate; N = near.

Need items used the verbal response categories « yes » (1) and « No » (2).

Wear and function items used the verbal response categories: « all the time » (1), « most of the time » (2), « some of the time » (3), « a little of the time » (4), « none of the time» (5).

Total spectacle independence for far vision was 100% in all groups. Only one patient in the ICB00 group was still wearing glasses all the time as she was used to it. All patients in all groups declared to be comfortable without glasses for distance vision “all the time”. Those percentage decreased to 80%, 75% and 70% respectively in the ICB00, PCB00 and MM for intermediate vision “all the time” In ICB00, PCB00 and MM, 100%, 80%, and 85% respectively consider they don‘t need glasses for intermediate vision. (Figure 4)

Regarding near vision, only 10% of patients declared never wearing glasses for reading in the ICB00 group, none of which belonged to PCB00 or MM. Moreover, respectively 30%, 16% and 15% in ICB00, PCB00 and MM declared they would be able to function comfortably without glasses for near vision.

## Discussion

In this study, we assessed post-cataract surgery patients for distance, intermediate and near visual acuity, comparing two IOLs from the same company: PCB00 vs ICB00. We could demonstrate that in patients with a bilateral Snellen visual acuity of 1.0 for distance vision without correction, the new ICB00 IOL lens could help increase intermediate visual acuity. Although this result could be expected, we have also shown an increase in near visual acuity.

Patients with the ICB00 IOL had a monocular one-line gain for CIVA, uncorrected near visual acuity (UNVA) and CNVA. Furthermore, this gain remained even in the assessment of binocular vision for CIVA and CNVA. Initial studies on ICB00 IOL did not show such a difference.^10^ Indeed, only corrected binocular intermediate vision was enhanced with the ICB00 IOL.

Those results are in line with recent studies,.^10–15^ that also found a gain in monocular intermediate vision in selected patients.

The tendency of ICB00 IOL patients’ group to have a better near monocular vision was not revelled in Menucci study. This might be due to a larger number of patient having a post-operative SE between −0.14 and + 0.14 rather than −0.5 and 0 as in our ICB00 group. A recent work from Sonam et al showed a significant increase in UNVA if compared to a PCB00 IOL. However, their ICB00 IOL group had a myopic post-operative SE, whereas their PCB00 IOL group did not, making the uncorrected comparison less relevant.^16^

Moreover, although non significant, we showed a gain of 2 lines in the binocular UNVA between PCB00 and ICB00 groups (0.3 vs 0.1 LogMAR). These results are encouraging, and a larger cohort of patients might confirm this tendency.

Regarding the mismatch group, our small number of patients did not enable us to detect significant improvement, the MM group had a similar pattern as the PCB00 group with a tendency to have a better near vision. Though results were quite heterogeneous with some gain in UNVA (PCB00 vs MM: 0.3 vs 0.1 p = 0.843), that disappeared with the use of optical correction. Those results are both surprising and unpublished to our knowledge. However, confirmation with a larger cohort of patients is needed.

Even though we could retrieve an improvement in near visual acuity with the ICB00 IOL, with up to 10% of patients implanted reporting a complete spectacles-independence for reading, some authors consider that an EDOF lens, such as the Tecnis Symfony provides a better near vision.^17^ Indeed, in their study, near vision was only 0.4 LogMAR whereas it reached 0.3 LogMAR in our study.

Our results in near vision are in accordance with a recent work comparing the Symfony IOL (Johnson&Johnson vision) with ICB00 IOL and concluding to a comparable clinical performance as binocular UNVA were similar.^18^

Nevertheless, debate still remains open between EDOF and ICB00 for near vision. Indeed, ICB00 IOLs advantages include their wide indication range to almost every patient as they can achieve a better optical quality, with less glare and halos.^19^

Patients comfort without glasses was similar in all three groups for distance and intermediate vision. Nevertheless, patients in the ICB00 IOL group needed glasses less often for intermediate and near activities when compared to the PCB00 IOL group, while patients in the MM group were in between the ICB00 IOL and the PCB00 IOL groups. 100% of ICB00 group patients were glasses-independent for intermediate vision, whereas, only 80 to 85%% were in the other groups. These results are in line with those of Mencucci et al.^10^ Also, 10% in the ICB00 group and 15% in the MM group were completely independent of glasses for reading. Nevertheless, the majority of patients in the ICB00 IOL and MM groups declared they occasionally did not use glasses for reading. This proportion was only 16% in the PCB00 IOL group. Those results are in accordance with those of Diogo and al. Indeed, they have found a greater capability for intermediate distance activities (i.e., daily life activities such as: computer use or price tags reading).^20^ One patient in the ICB00 IOL group was still wearing glasses because she was used to wearing glasses since her childhood, even though she declared being able to function comfortably without glasses most of the time, reflecting the importance of patient expectations and habits.

Although ICB00 IOLs are not designed for reading, the central defocus helps patients achieve some degree of spectacle independence, as we could show in our study. Thus, in our study, bilateral implantation with ICB00 IOLs gave a 100% spectacle-independence for intermediate vision and also a moderate help for reading. The main advantage is that this gain does not cost any compromise in terms of distance vision, nor in terms of halos and glares as previously discussed.

Given the retrospective nature of the study, patients were not selected based on their pupil size in the pre-operative settings. It is now known that pupil size needs to be around 2 mm for near vision, so it is possible that we were not able to obtain the best intermediate vision from ICB00 IOLs. Indeed, for pupils larger than 3.5 mm, PCB00 and ICB00 IOLs are comparable.^8^ In addition, the sample size of the MM group was small, leading to a lower chance to detect any statistically significant difference. One strength of this study was that the post-operative visual acuity was assessed by an optometrist in a prospective and blinded fashion. As patients were not selected in a prospective manner prior to surgery, we had a significant difference in the mean pre-operative corneal Spherical-Equivalent. As the MM group had the highest and the ICB00 the lowest Spherical-Equivalent, the PCB00 being in-between, it is hard to conclude if it has an impact on the intermediate or near vision, but it has to be considered.

## Conclusions

We selected patients with a goal outcome of a binocular 1.0 Snellen visual acuity without correction (which means spectacle-free for far vision), to determine if, in this ideal condition, the monocular or binocular implantation of an ICB00 IOL would be of any benefit.

We retrieved a tendency towards better intermediate and near vision by one line, which was statistically significant with proper distance correction. This one-line gain had an impact on patient‘s quality of life, as patients in the ICB00 IOL group need for spectacles was lower than patients in the PCB00 IOL group. If one patient is implanted in one eye with a monofocal IOL, he might still experience an improvement in near vision if the other eye is implanted with the ICB00 IOL.

## Supplemental Material

sj-docx-1-ejo-10.1177_11206721221146675 - Supplemental material for Real world outcomes of the new Tecnis Eyhance IOL Click here for additional data file. Supplemental material, sj-docx-1-ejo-10.1177_11206721221146675 for Real world outcomes of the new Tecnis Eyhance IOL by Edward Gigon, Walid Bouthour, Georgios D Panos, Bojan Pajic and Horace Massa in European Journal of Ophthalmology
