# Supplementary material for: Real world outcomes of the new Tecnis Eyhance IOL
Source: Eur J Ophthalmol. 2022 Dec 25;33(3):1390–7. doi: 10.1177/11206721221146675 (PMC10152560; doi:10.1177/11206721221146675)

Supplementary file 1: post-operative repartition of the spherical equivalent in diopter between the 3 groups.


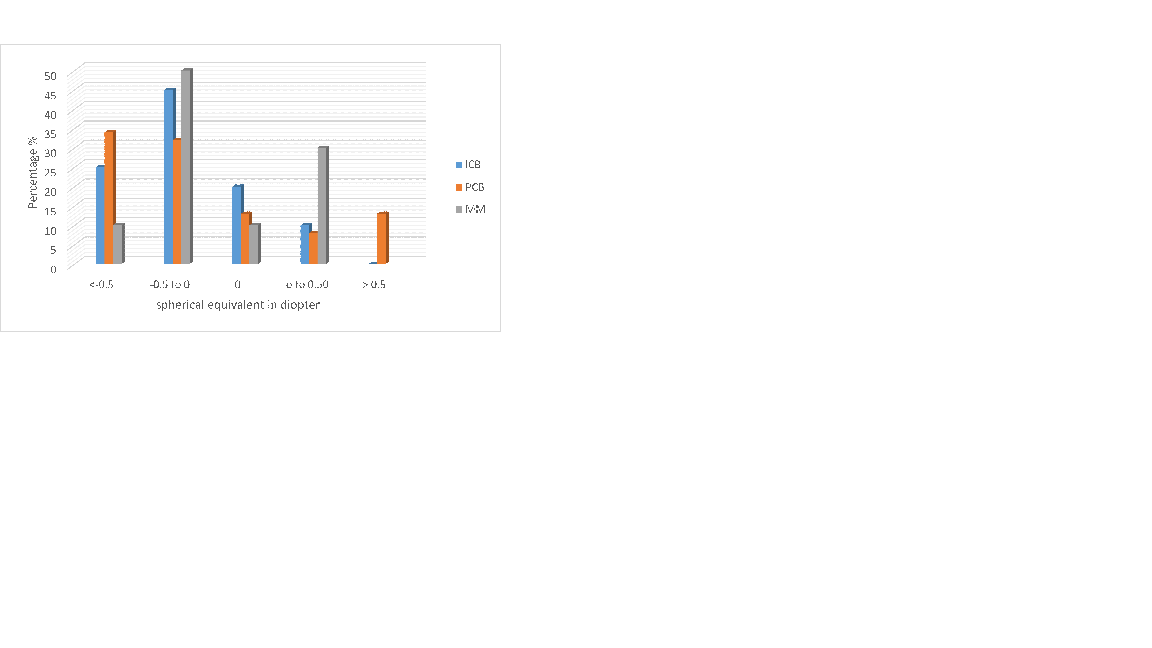

Supplement: sj-docx-1-ejo-10.1177_11206721221146675 - Supplemental material for Real world outcomes of the new Tecnis Eyhance IOL [file sj-docx-1-ejo-10.1177_11206721221146675.docx]
